# Respiratory Drive in Patients with Sepsis and Septic Shock: Modulation by High-flow Nasal Cannula

**DOI:** 10.1097/ALN.0000000000004010

**Published:** 2021-10-13

**Authors:** Tommaso Mauri, Elena Spinelli, Bertrand Pavlovsky, Domenico Luca Grieco, Irene Ottaviani, Maria Cristina Basile, Francesca Dalla Corte, Gabriele Pintaudi, Eugenio Garofalo, Annalisa Rundo, Carlo Alberto Volta, Antonio Pesenti, Savino Spadaro

**Affiliations:** From the Department of Anesthesia, Critical Care and Emergency, Institute for Treatment and Research, Ca’ Granda Maggiore Policlinico Hospital Foundation, Milan, Italy (T.M., E.S., B.P., M.C.B., A.P.); the Department of Pathophysiology and Transplantation, University of Milan, Milan, Italy (T.M., A.P.); the Department of Anesthesiology and Intensive Care Medicine, Catholic University of the Sacred Heart, Fondazione Policlinico Universitario A. Gemelli Institute for Treatment and Research, Rome, Italy (D.L.G., G.P.); the Intensive Care Unit, Department of Morphology, Surgery and Experimental Medicine, Sant’Anna University Hospital, Ferrara, Italy (I.O., F.D.C., C.A.V., S.S.); the Department of Medical and Surgical Science, Università Magna Graecia, Catanzaro, Italy (E.G.); the Department of Anesthesiology and Intensive Care Medicine, Polo Ospedaliero Belcolle ASL, Viterbo, Italy (A.R.).

## Abstract

Experimental and pilot clinical data suggest that spontaneously breathing patients with sepsis and septic shock may present increased respiratory drive and effort, even in the absence of pulmonary infection. The study hypothesis was that respiratory drive and effort may be increased in septic patients and correlated with extrapulmonary determinant and that high-flow nasal cannula may modulate drive and effort.

**Methods:**

Twenty-five nonintubated patients with extrapulmonary sepsis or septic shock were enrolled. Each patient underwent three consecutive steps: low-flow oxygen at baseline, high-flow nasal cannula, and then low-flow oxygen again. Arterial blood gases, esophageal pressure, and electrical impedance tomography data were recorded toward the end of each step. Respiratory effort was measured as the negative swing of esophageal pressure (ΔP_es_); drive was quantified as the change in esophageal pressure during the first 500 ms from start of inspiration (P_0.5_). Dynamic lung compliance was calculated as the tidal volume measured by electrical impedance tomography, divided by ΔP_es_. The results are presented as medians [25th to 75th percentile].

**Results:**

Thirteen patients (52%) were in septic shock. The Sequential Organ Failure Assessment score was 5 [4 to 9]. During low-flow oxygen at baseline, respiratory drive and effort were elevated and significantly correlated with arterial lactate (*r* = 0.46, *P* = 0.034) and inversely with dynamic lung compliance (*r* = –0.735, *P* < 0.001). Noninvasive support by high-flow nasal cannula induced a significant decrease of respiratory drive (P_0.5_: 6.0 [4.4 to 9.0] *vs*. 4.3 [3.5 to 6.6] *vs*. 6.6 [4.9 to 10.7] cm H_2_O, *P* < 0.001) and effort (ΔP_es_: 8.0 [6.0 to 11.5] *vs*. 5.5 [4.5 to 8.0] *vs*. 7.5 [6.0 to 12.6] cm H_2_O, *P* < 0.001). Oxygenation and arterial carbon dioxide levels remained stable during all study phases.

**Conclusions:**

Patients with sepsis and septic shock of extrapulmonary origin present elevated respiratory drive and effort, which can be effectively reduced by high-flow nasal cannula.

## Abstract 2

Respiratory drive and effort and dynamic lung compliance were evaluated in 25 nonintubated patients with extrapulmonary sepsis or septic shock using arterial blood gases, esophageal pressure monitoring, and electrical impedance tomography at baseline with low-flow nasal oxygen therapy, during high-flow nasal cannula support and again with low-flow nasal oxygen therapy. Patient comfort was evaluated using a 10-point visual analog scale at each step. High-flow nasal oxygen therapy significantly reduced elevated respiratory drive and effort. There was no correlation between patient perceived comfort and measures of drive and effort. The impact of the findings from this physiologic study on patient outcome remain to be determined.

Supplemental Digital Content is available in the text.

**Editor’s Perspective**

**What We Already Know about This Topic**

Increases in respiratory drive and effort in critically ill patients may place the patient at higher risk for respiratory failure and intubation.

The authors have previously shown that respiratory drive and effort are significantly increased in patients with pulmonary infection and that support by high-flow nasal cannula significantly reduces this increase relative to low-flow oxygen therapy.

Whether respiratory drive is increased and the effect of high-flow nasal cannula in patients with extrapulmonary sepsis remain unknown.

**What This Article Tells Us That Is New**

Respiratory drive and effort and dynamic lung compliance were evaluated in 25 nonintubated patients with extrapulmonary sepsis or septic shock using arterial blood gases, esophageal pressure monitoring, and electrical impedance tomography at baseline with low flow nasal oxygen therapy during high-flow nasal cannula support and again with low-flow nasal oxygen therapy. Patient comfort was evaluated using a 10-point visual analog scale at each step.

High-flow nasal oxygen therapy significantly reduced elevated respiratory drive and effort.

There was no correlation between patient perceived comfort and measures of drive and effort.

The impact of the findings from this physiologic study on patient outcome remain to be determined.

Sepsis and septic shock are deadly syndromes characterized by intense acute inflammatory reaction.^1^ Mediators produced at the site of infection are poured into systemic circulation and activate amplification pathways within and between peripheral target organs.^2^

Proinflammatory stimuli to the central nervous system trigger an increase in body temperature.^3^ Activation of the sympathetic response and release of stress hormones increase the cardiovascular tone.^4^ These responses alter the metabolic demands of the organism, increasing carbon dioxide production.^5,6^ A compensatory increase of the respiratory drive will be the price to pay to eliminate sepsis-induced excess of carbon dioxide through adequate minute ventilation.^7^

Metabolic acidosis due to poor peripheral perfusion, lactate production, impaired renal function, and altered plasma buffers will further increase minute ventilation to compensate for systemic acidosis.^8^ Moreover, spontaneously breathing septic patients often present with respiratory alkalosis, because arterial carbon dioxide levels fall below the compensatory value. This is likely due to further activation of the respiratory drive by inflammatory stimuli targeting central and peripheral chemosensors and generating exaggerated breathing response.^9^

Although there is scant data, if any, on human subjects,^10^ the authors reasoned that activation of the above-mentioned mechanisms (*i.e.*, increased metabolic activity, metabolic acidosis, inflammatory mediators) could lead to increased respiratory drive, resulting in excessive inspiratory effort in sepsis and septic shock patients, even in the absence of pulmonary infection.

Relevant clinical consequences of increased respiratory drive during sepsis and septic shock could be many: increased muscular effort poses the risk of diaphragm fatigue and pump failure^11^; higher inspiratory transpulmonary pressure may lead to patient self-inflicted lung injury in lungs already “hit” by soluble inflammatory mediators^12^; the increase in oxygen consumption by the respiratory muscles could further impair the delivery/consumption imbalance and precipitate cardiovascular failure.^13^ Two large clinical studies already showed extremely high mortality of spontaneously breathing septic patients intubated during their intensive care unit (ICU) stay *versus* those patients who were never intubated.^14,15^ All these data generate the hypothesis that modulation of respiratory drive and effort might represent a relevant physiologic goal in spontaneously breathing patients with sepsis and septic shock in the ICU.

In patients with acute hypoxemic respiratory failure, high-flow nasal cannula improves clinical outcomes through multiple physiologic mechanisms (*e.g.*, decreased effort, dead space wash-out, increased alveolar Fio_2_, and improved comfort).^16–18^ Even though these mechanisms may also be beneficial in patients with increased drive caused by extrapulmonary causes, they have not been evaluated in patients with sepsis and septic shock without pneumonia. The aim of this study was to measure respiratory drive and effort in these patients and to assess the physiologic effects of high-flow nasal cannula. The study hypothesis was that respiratory drive and effort may be increased in septic patients and correlated with extrapulmonary determinants (*e.g.*, metabolic acidosis) and that high-flow nasal cannula may modulate drive and effort.

## Materials and Methods

### Patient Population

Between March 2019 and November 2020, 25 nonintubated patients admitted to 3 ICUs in Italy with a diagnosis of sepsis or septic shock were enrolled. Sepsis and septic shock were defined according to the Sepsis-3 consensus guidelines.^1^ The exclusion criteria were diagnosis of pneumonia, severe chronic obstructive pulmonary disease, contraindication to the use of an esophageal balloon catheter, and encephalopathy with a Glasgow coma scale score of less than 12.

This study was approved by the ethical committees of each participating center (promoting and coordinating center: Maggiore Policlinico Hospital, Milan, Italy; reference No. 193_2019bis). Written informed consent was obtained from all participants before enrollment. The study was planned and conducted according to ethics and transparence guidelines following the Declaration of Helsinki. Because this was an explorative physiologic study, the methods used were not registered on a public server before its completion, as for other similar studies in this field.^17–20^

### Clinical Data

After enrollment, the following characteristics were recorded: age, height, weight, body mass index, length of stay in the ICU before inclusion, oxygenation under clinical respiratory support (*i.e.*, Pao_2_/Fio_2_ ratio), clinical severity assessed by SAPS II and Sequential Organ Failure Assessment (SOFA) scores, plasma lactate and C-reactive protein levels, and use of vasopressors.

### Monitoring

An esophageal balloon catheter (Cooper Surgical, USA) was inserted through the nose, inflated following the manufacturer’s recommendations, and secured. Appropriate positioning was confirmed by insertion depth, presence of cardiac artifacts, and convincing inspiratory swings. The esophageal pressure (P_es_) signal was recorded intermittently with a dedicated acquisition system at a 100-Hz sample rate and analyzed offline.

An electrical impedance tomography belt was placed between the fourth and fifth intercostal space and connected to its recording device. The acquisition sample rate was set at 50 Hz. The electrical impedance tomography data were continuously acquired and analyzed offline with dedicated software. Detailed information about the devices and software used in each center is available in the Supplemental Digital Content (table A1, http://links.lww.com/ALN/C699).

### Study Protocol

Patients were kept in a semirecumbent position without sedation. Adequate analgesia was checked before the start of the protocol (visual analog scale [VAS] of 3 or lower). A calm environment was ensured around the patients throughout the study. Each patient underwent 3 consecutive 30-min steps:

1) Low-flow oxygen–baseline, with no support or low-flow oxygenation device to maintain peripheral oxygen saturation (Spo_2_) at greater than 94%
2) High-flow nasal cannula, with flow 50 l/min, temperature 34 to 37°C and Fio_2_ to maintain Spo_2_ at greater than 94%
3) Low-flow oxygen–end, same support and settings as during the low-flow oxygen–baseline step.

During Steps 1 and 3, low-flow oxygen support was delivered through nasal cannula or standard nonocclusive facemask, according to the clinical practice of each center (Supplemental Digital Content, table A2, http://links.lww.com/ALN/C699). In Step 2, high-flow nasal cannula was provided through a dedicated system (Airvo 2, Fisher & Paykel Healthcare, New Zealand).

Toward the end of each step, the following was recorded: respiratory rate (RR), Spo_2_, mean arterial pressure, and heart rate. Then a 3-min recording of the P_es_ waveform was stored, and arterial blood gas was measured. During the high-flow nasal cannula step, the Fio_2_ was assessed directly by the device, while the Fio_2_ during the two low-flow oxygen steps was calculated as follows: [21 + (O_2_ flow in l/min × 3)]%.^21^

The ratio of oxygen saturation was computed as the Spo_2_/Fio_2_ ratio divided by RR.^22^ Patients were asked to rate the comfort related to each respiratory support by VAS, ranging between 0 (extreme discomfort) to 10 (very comfortable).

### Esophageal Pressure

Data from esophageal pressure waveforms from 10 consecutive representative breaths were computed offline from recordings performed at the end of each step. All tracings were analyzed by two independent observers. In four patients, tracings were discarded due to poor quality of the waveforms (Supplemental Digital Content, fig. A1a and A1b, http://links.lww.com/ALN/C699).

Respiratory effort was assessed by maximal amplitude of P_es_ change during negative inspiratory swing (ΔP_es_). An estimate of the metabolic work of breathing was calculated as the esophageal pressure-time product per minute (Supplemental Digital Content, fig. A2, http://links.lww.com/ALN/C699)^17^.

### Electrical Impedance Tomography

Offline analysis of electrical impedance tomography data allowed the calculation of tidal volume (V_T_) by measuring the average tidal impedance variation (10 representative breaths recorded at the end of each step) and then converting arbitrary units into milliliters based on a calibration factor derived from a similar population of ICU patients from a previous study.^18^ Supplemental information about this calibration process is provided in the Supplemental Digital Content (http://links.lww.com/ALN/C699).

Minute ventilation was computed as the product of V_T_ × RR. Dynamic compliance of the lung was calculated as the ratio of V_T_/ΔP_es_, as previously described.^17^ Lung homogeneity was assessed by the ratio between tidal impedance variation in the ventral and dorsal regions (V_T-NDEP/DEP_).^17,18^

### Respiratory Drive

Respiratory drive was measured by three P_es_ and electrical impedance tomography–based measures: the inspiratory esophageal pressure change during the first 500 ms from the start of inspiration (P_0.5_)^23^; the slope of the inspiratory negative P_es_ swing from the start of inspiration to the minimum pressure (ΔP_es_/Δt); and the mean inspiratory flow, calculated as the ratio between V_T_ and the inspiratory time (V_T_/Ti).^24^ Through the use of these indexes, the authors aimed to assess the two dimensions of drive: the intensity (P_0.5_ and ΔP_es_/Δt) and the amplitude (V_T_/Ti).

### Statistical Analysis

All results are presented as median [interquartile range] or number (%). Distribution normality was checked for each variable using the D’Agostino–Pearson test. Based on previous studies, sample size calculation (n = 20) was performed by hypothesizing a change of ΔP_es_ of 2.5±3 cm H_2_O between study Steps 1 and 2,^17,18^ with a two-tailed type I error of 5% and statistical power of 80%. Because we predicted a feasibility for ΔP_es_ measurement of 80%, sample size was increased to 25 patients.

Comparisons of physiologic variables between the three study steps were preplanned and performed by using one-way repeated measures ANOVA on rank. *Post hoc* comparisons between the low-flow oxygen–baseline and the two following steps were performed by Dunnett’s test.

Correlations between ΔP_es_ and selected physiologic variables were performed by Spearman’s correlation. To further identify independent determinants of respiratory effort, we performed a multiple linear regression including the two factors significantly associated with ΔP_es_ at Spearman’s correlation (*i.e.*, ΔP_es_/V_T_ and arterial lactate) and adjusted for age, body mass index, and SOFA score. Spearman’s correlations were also used to assess the relationship between comfort scale (VAS), ΔP_es_, and P_0.5_.

The association between ΔP_es_ and measures of respiratory drive was assessed by linear regression, pooling data from all three study steps (n = 63). Measurements were assumed to be independent across individuals.

Given the amount of physiologic data that we measured in adjunct to esophageal pressure, all 25 patients were analyzed for main outcomes. Then we repeated the analyses only in the subgroup of patients with high-quality waveforms of esophageal pressure (n = 21). Finally, we performed a *post hoc* sensitivity analysis (*i.e.*, subgroup analysis) on nonhypoxemic patients (*i.e.*, patients with Pao_2_/Fio_2_ ratios of greater than 200 mmHg upon enrollment, n = 21) to avoid overlap with results from our previous study.^17^ For all analyses, a two-tailed *P* value of less than 0.05 was considered significant. Statistical analysis was performed using SPSS (SPSS Statistics, USA) and Prism (GraphPad version 9.0, USA).

## Results

Twenty-five patients were enrolled in this study. The main characteristics of the study population are described in table 1. The median age was 69 [interquartile range, 54 to 79] yr old. Seventeen patients (68%) were enrolled within 24 h from admission, with a median ICU stay before enrollment of 1 [0 to 2] day. The median SOFA score was 5 [4 to 9], and 13 patients (52%) were in septic shock (table 1).

**Table 1.**
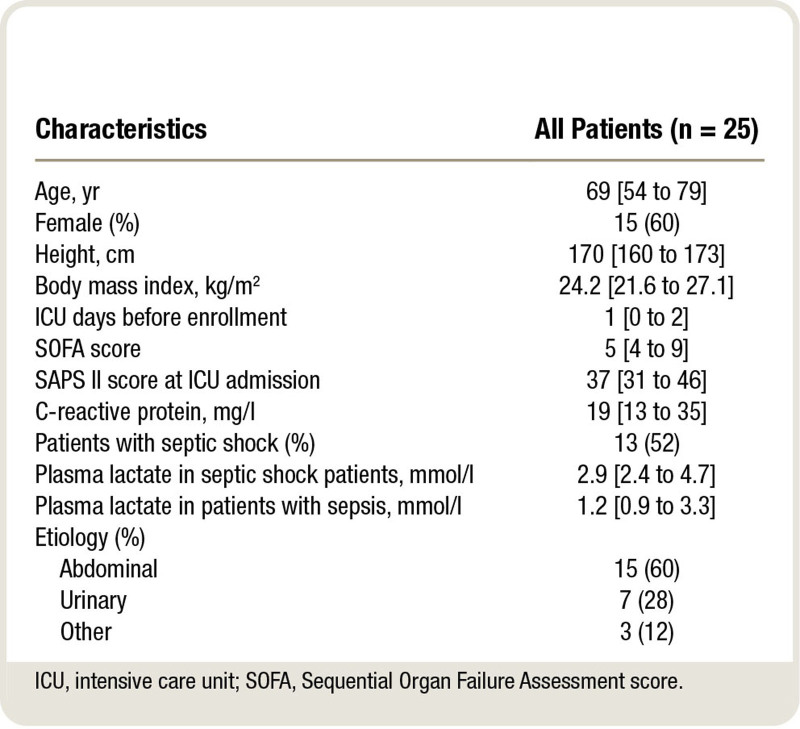
Patients’ Main Characteristics

As mentioned above, esophageal data were missing for four patients, because of poor quality of the recorded waveforms. Arterial blood gas analysis was not available for one patient during the low-flow oxygen–baseline step for technical reasons. There were no other missing data.

**Figure 1.**
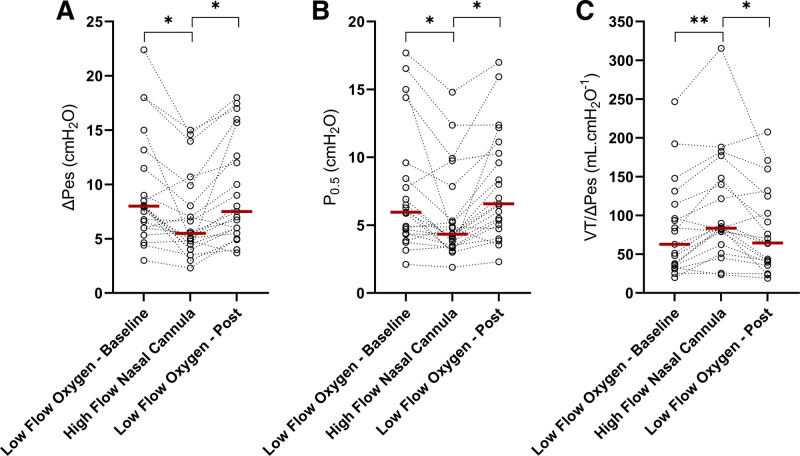
Effects of high-flow nasal cannula on negative esophageal pressure swing (patient’s effort: ΔP_es_, *A*); ΔP_es_ at 500 ms from the start of effort (patient’s drive: P_0.5_, *B*); and dynamic lung compliance (V_T_/ΔP_es_, *C*). *Red bars* indicate median value. *P* values for the ANOVA test are reported in table 2. *Post hoc* Dunnett’s *P* values: *P < 0.005; **P < 0.05.

Respiratory effort assessed by ΔP_es_ and was elevated during low-flow oxygen–baseline and low-flow oxygen–end phases and significantly decreased during the high-flow nasal cannula phase, in comparison to both low-flow oxygen steps (ANOVA *P* < 0.001; table 2; fig. 1A). Support by high-flow nasal cannula was also associated with a decrease of respiratory drive: all three variables that were measured as surrogate of central drive (P_0.5_, ΔP_es_/Δt, and V_T_/Ti) significantly fell during high-flow nasal cannula (*P* < 0.01 for all; table 2; fig. 1B). Of note, high-flow nasal cannula modulated respiratory drive and effort even in patients with relatively normal values during the first low-flow oxygen step (fig. 1). The absolute changes in respiratory effort and drive between study phases were so relevant that they may be considered clinically significant (table 2).

**Table 2.**
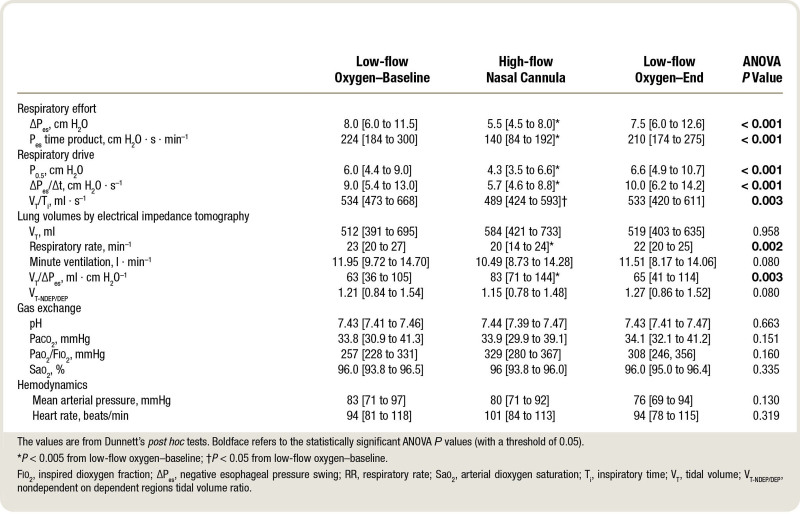
Physiologic Effects of High-flow Nasal Cannula in Spontaneously Breathing Patients with Sepsis and Septic Shock

Electrical impedance tomography allowed noninvasive assessment of minute ventilation and dynamic lung compliance (V_T_/ΔP_es_; table 2; fig. 1C). During high-flow nasal cannula, minute ventilation decreased (*P* = 0.080) with unchanged arterial carbon dioxide levels (*P* = 0.151), and the V_T_/ΔP_es_ ratio improved (*P* = 0.003). Lung homogeneity assessed by electrical impedance tomography with the V_NDEP/DEP_ ratio improved (*P* = 0.080) as well (table 2).

As expected by study protocol, there was no difference between the three phases in terms of Spo_2_ (*P* = 0.335), and the Pao_2_/Fio_2_ ratio did not change either (*P* = 0.160; table 2). Hemodynamics remained stable during high-flow nasal cannula (table 2). All the main study comparisons presented in table 2 were reanalyzed in the subgroups of nonhypoxemic patients and those with high-quality esophageal pressure tracings (n = 21 for both, see “Materials and Methods” above), leading to similar results (Supplemental Digital Content, tables A3 and A4, http://links.lww.com/ALN/C699).

Although maybe relevant only for hypoxemic patients, the authors measured the ratio of oxygen saturation, which increased during the high-flow nasal cannula phase (low-flow oxygen–baseline 11.7 [9.4 to 17.1] *vs.* high-flow nasal cannula 18.5 [15.6 to 25.6] *vs.* low-flow oxygen–end 14.8 [11.9 to 19.4]; *P* < 0.001). High-flow nasal cannula was well tolerated: comfort assessed by the VAS scale did not differ between the three phases (low-flow oxygen–baseline 8 [7 to 9] *vs*. high-flow nasal cannula 8 [7 to 9] *vs.* low-flow oxygen–end 8 [8 to 9]; *P* = 0.119). Of note, there was no correlation between ΔP_es_, P_0.5_, and the comfort VAS during the low-flow oxygen–baseline step (Supplemental Digital Content, fig. A3, http://links.lww.com/ALN/C699).

**Figure 2.**
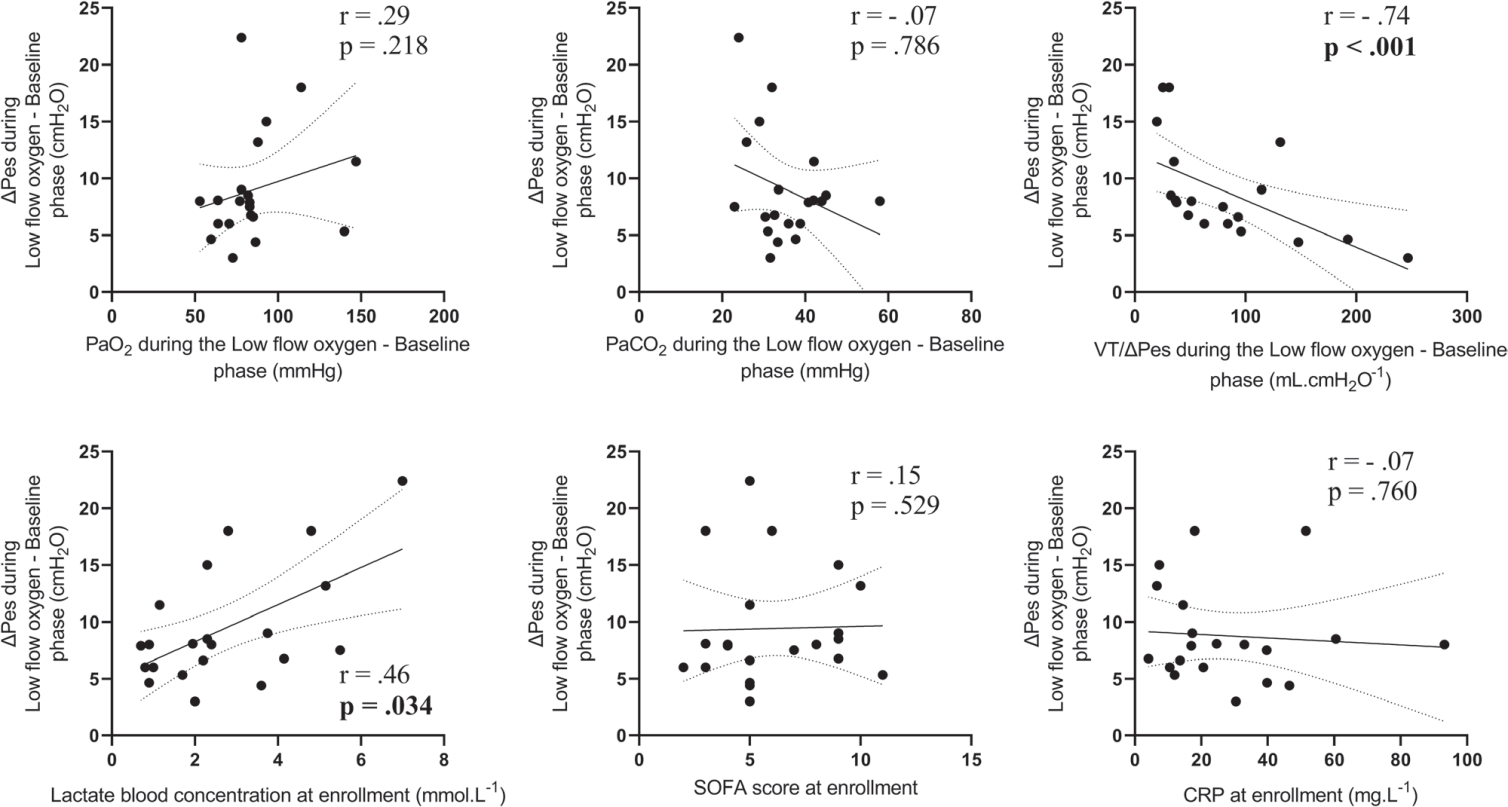
Correlations between physiologic determinants of inspiratory effort *versus* ΔP_es_ during the low-flow oxygen–baseline step. Spearman’s correlation was computed for each variable. r and *p* values are reported in the figure for each variable.

To explore the main determinants of increased inspiratory effort during sepsis and septic shock, the authors evaluated the correlation between ΔP_es_ assessed within the low-flow oxygen–baseline phase and physiologic respiratory stimuli measured at the bedside. Figure 2 shows all the correlations: ΔP_es_ was significantly associated with arterial lactate and inversely with the V_T_/ΔP_es_ ratio (*r* = 0.46, *P* = 0.034 and *r* = –0.76, *P* < 0.001, respectively). Interestingly, altered arterial blood gases (O_2_ and carbon dioxide levels), sepsis severity (SOFA score), and biomarker of inflammation (C-reactive protein) did not correlate with respiratory effort. In the multivariate model adjusted for clinical confounders (see the “Materials and Methods” above), both arterial lactate (β-coefficient: 1.70 [95% CI 0.53 to 2.87], *r*^2^ = 0.41, *P* = 0.012) and the V_T_/ΔP_es_ ratio (β-coefficient: –0.05 [–0.08 to –0.02], *r*^2^ = 0.39, *P* = 0.012) were independently correlated with ΔP_es_.

**Figure 3.**
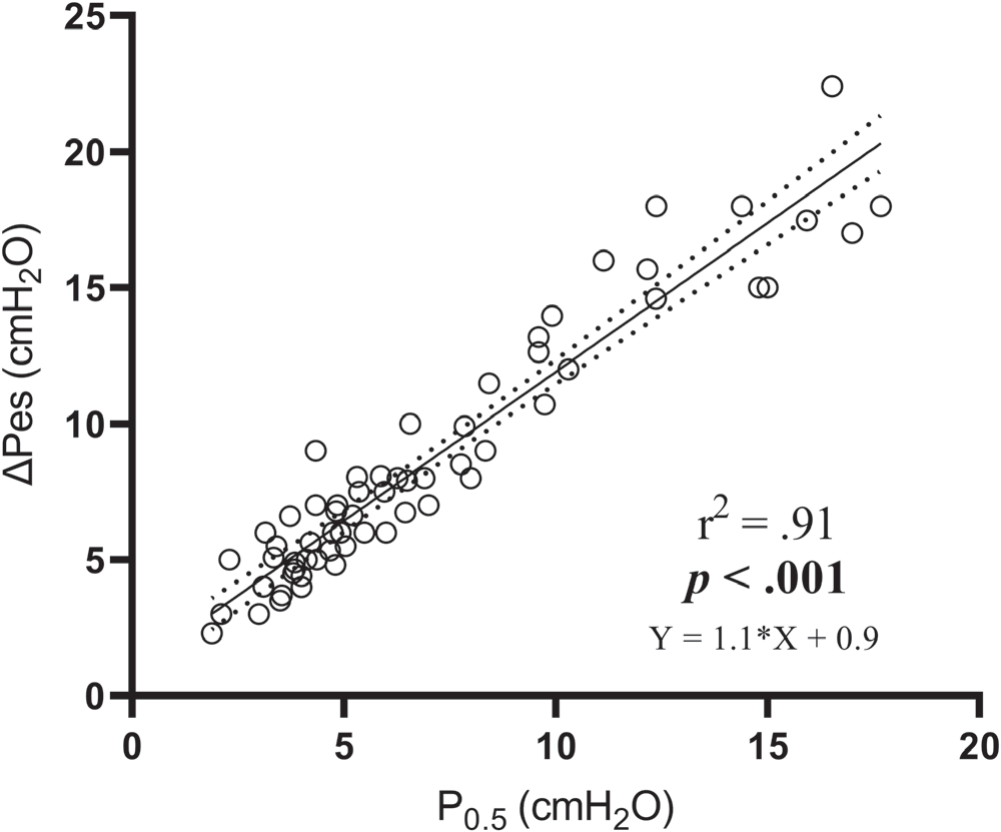
Dunnett’s *post hoc* tests: *P < 0.05 from low-flow oxygen–baseline; †*P* < 0.005 from low-flow oxygen–baseline. Data from all three study steps were gathered. Linear regression values are provided with 95% CIs and equation.

The correlation between drive and effort may be lost in the presence of neuromuscular insufficiency, but in the current study performed early after admission, the correlation between P_0.5_ and ΔP_es_ was statistically significant (*r* = 0.95; *P* < 0.001; fig. 3). Significant correlations existed also between ΔP_es_ and ΔP_es_/Δt, whereas correlation with mean inspiratory flow was poorer (Supplemental Digital Content, fig. A4, http://links.lww.com/ALN/C699). ΔP_es_ during the high-flow nasal cannula phase improved more in patients with higher baseline RR and ΔP_es_, with a linear relationship between these variables and the ΔP_es_ improvement (Supplemental Digital Content, fig. A5, http://links.lww.com/ALN/C699).

## Discussion

The main findings of the study can be summarized as follows: respiratory drive and effort are increased in patients with sepsis and septic shock of extrapulmonary origin, and noninvasive support by high-flow nasal cannula modulates them effectively; higher plasma lactate level and lower dynamic lung compliance are associated with more intense respiratory effort; respiratory drive and effort are tightly correlated early after admission to the ICU; and higher inspiratory effort and respiratory rate during low-flow oxygen are associated with more effective modulation of effort by high-flow nasal cannula. This study confirmed the hypothesis of increased respiratory effort in patients with sepsis and septic shock in comparison to controls. The median for ΔP_es_ of 8.0 cm H_2_O measured in septic patients is clearly higher than 3.2 cm H_2_O measured in recent series on healthy adults.^25^ In addition, more than 75% of septic patients had pressure-time product values above the physiologic upper threshold of 150 cm H_2_O · s · min^-1^.^26^ Experimental and pilot clinical data showed that, during sepsis, multiple mechanisms could lead to increased respiratory drive and effort.^10,27^

Askanazi *et al.*^5^ showed that infusion of catecholamines and stress hormones (which are hallmark mediators of the systemic septic syndrome) increase the O_2_ consumption and carbon dioxide production in healthy subjects, leading to a compensatory increase of minute ventilation. Metabolic acidosis is highly prevalent in sepsis and septic shock; the etiology is multifactorial, and the severity of the acidosis is correlated with the outcome.^28^ Metabolic acidosis is accompanied by respiratory compensation and hyperventilation to clear carbon dioxide, if the patient is able to manage that.^8,10^

Tang *et al.*^9^ showed that an intravenous challenge with 50 mg/kg endotoxin in healthy rats increases the minute ventilation by 144% within 5 h and that tachypnea is prevented by vagotomy, suggesting that hyperventilation is mediated by lung vagal afferents. Interestingly, in that study, the effects of endotoxin were independent from alterations of gas exchange.^9^ Similarly, Huxtable *et al.*^29^ described increased respiratory rates in rats treated with lipopolysaccharide and hypothesized a direct action on the brainstem centers. Finally, the study findings may resemble experimental human data by Doorduin *et al.*^30^: Lipopolysaccharides infused in healthy humans induced an increase in diaphragmatic strength, which might explain the tight correlation with drive.

In summary, metabolic demands, acidosis, and inflammation increase the respiratory drive and, if muscular function is preserved, the effort in septic patients.^10,31^ The close correlation between drive and effort that we describe likely suggests that the muscular function in the patient population was not impaired, and higher drive directly produced an increase in ventilation. Previous clinical study confirmed a more rapid and shallow breathing pattern in intubated septic patients during weaning, which is a quite different setting in comparison with the current data.^27^ Septic patients may be considered at risk of increased respiratory drive and effort, even when their lungs are not the primary site of infection.

To further describe the correlation between determinants of drive and the respiratory effort in the study population, the authors correlated markers of each mechanism with ΔP_es_. Plasma lactate and dynamic lung compliance were the only factors showing an association. Lactate is one of the main determinants of cerebrospinal fluid acidosis, which directly stimulates the respiratory centers^32^; however, they are also correlated with the overall severity of sepsis, and a simple noncausal association cannot be excluded.^33^ Dynamic compliance may be a more sensitive indicator of the impairment in lung function than gas exchange, and although patients with pneumonia were excluded from the study, initial lung injury caused by circulating mediators and/or high lung stress may have already been at play to increase respiratory drive and effort.

In our population, there was no association between the respiratory drive and effort and the comfort perceived by the patient, making this measure unsuitable as a marker of increased transpulmonary pressure. This finding likely highlights the very complex interaction between acute respiratory failure and comfort: other determinants like mucosal dryness, pain, psychologic stress, and fear might become predominant over more specific mechanisms such as increased respiratory load. Similar lack of linear correlation between respiratory pattern and comfort was already described in patients with acute exacerbation of chronic obstructive pulmonary disease.^34^

Previously published data on patients with acute respiratory hypoxemic failure (including only patients with Pao_2_/Fio_2_ of less than 200, 87% with pneumonia) already showed a decrease in respiratory effort by noninvasive support with high-flow nasal cannula.^17^ In the current study, only 4 patients (16%) had Pao_2_/Fio_2_ of less than 200, and pneumonia was an exclusion criterion. Despite different settings and minimum overlap with previous study, high-flow nasal cannula modulated both the intensity and the amplitude of respiratory drive of septic patients, and this likely decreased the instantaneous per-breath effort (ΔP_es_), as well as the surrogate index of longer term work of breathing (pressure-time product). The most probable mechanisms leading to beneficial effects of high-flow nasal cannula in septic patients may have been washout of the dead space, compensating excessive carbon dioxide production, and some expiratory positive pressure effect, improving dynamic lung compliance.^17,18^ Regardless of the mechanism used for the same clinical condition, by application of high-flow nasal cannula, the muscles of septic patients may have to bear less work of breathing, and their lungs may be subject to decreased lung stress.

The potential clinical impact of the authors’ findings is correlated with the risk of worse clinical outcome in spontaneously breathing septic patients intubated during their ICU stay. In a *post hoc* analysis of data from a large randomized clinical trial on 776 septic shock patients, Delbove *et al.*^14^ described significantly lower mortality in patients admitted to the ICU and never requiring intubation *versus* those intubated during their ICU stay. Later on, the prospective INTUBATIC study performed by Darreau *et al.*^15^ enrolled 859 spontaneously breathing patients with septic shock admitted to the ICU: in-hospital mortality was 46.9% in patients intubated within 8 h *versus* 41.2% in patients intubated between 8 and 72 h and 13.1% in patients who were never intubated. Interestingly, in that study, use of accessory muscles (likely indicating strong inspiratory effort) and higher respiratory rate characterized patients who ended up intubated *versus* those who were never intubated. Respiratory effort and rate are the same factors that, in the current study, correlated with larger reduction of effort by high-flow nasal cannula. However, the primary endpoint of the current study was physiologic.

This study presents some limitations. First, findings should be generalized with caution because of the limited number of patients enrolled and given the heterogeneous nature of sepsis. However, the sample size is larger than previous physiologic studies on the same topic.^17,18,20,27^ Second, esophageal pressure measurements were performed without calibration according to the method described by Baydur *et al.*,^35^ but the current study used objective criteria to evaluate the quality of online measures (insertion depth, cardiac artifacts, amplitude of negative P_es_ swings). Third, electrical impedance tomography was calibrated by a factor derived from another patient population with similar characteristics (adult nonintubated critically ill patients),^18^ for whom synchronized spirometry and electrical impedance tomography data were collected; although the factor may not be as accurate for this population, the relative effects between study steps should be reliable. Fourth, the authors did not assess the differential role of the diaphragm *versus* accessory inspiratory and expiratory muscles (*e.g.*, by ultrasonography)^36^ as the origin of increased effort. Of note, experimental studies in dogs exposed to endotoxin showed homogeneous activation of all the inspiratory muscles.^37,38^ In a cohort of intubated septic patients, use of accessory muscles measured by ultrasonography was verified in the majority of cases.^36^ These findings generate the hypothesis that both the diaphragm and the accessory muscles may generate increased effort in septic patients, and their differential role deserves further exploration. Fifth, the correlation between ΔP_es_ and dynamic lung compliance (V_T_/ΔP_es_) may suffer by some degree of mathematical coupling. However, we present it given the sound physiologic background linking respiratory mechanics and effort.^39^ Sixth, the three measures of respiratory drive may have been influenced by respiratory muscle strength and/or respiratory system compliance, and conclusions regarding the impact of high-flow nasal cannula on respiratory drive in septic patients should be taken with caution. Finally, both sepsis and septic shock patients were enrolled in this study, which may have introduced some heterogeneity; the authors wanted to explore a spectrum of severity to analyze correlations between clinical and biochemical factors and respiratory drive and effort.

### Conclusions

High-flow nasal cannula modulates effectively elevated respiratory drive and effort in patients with sepsis and septic shock of extrapulmonary etiology. Higher lactatemia and lower dynamic lung compliance characterize patients with stronger inspiratory effort. Higher respiratory rate and effort during low-flow oxygen may predict larger modulation of effort by high-flow nasal cannula. The study findings generate the hypothesis that noninvasive respiratory support by high-flow nasal cannula in septic patients without pneumonia might reduce the risks of increased inspiratory effort.

### Research Support

Supported by departmental funding from Ricerca Corrente, Ospedale Maggiore Policlinico, Milan, Italy, and by an unrestricted research grant by Fisher & Paykel Healthcare, Auckland, New Zealand.

### Competing Interests

Dr. Mauri received personal fees from Drager (Lubeck, Germany), Fisher & Paykel Healthcare (Auckland, New Zealand), Mindray (Nanshan, China), and BBraun (Melsungen, Germany), all outside of the submitted work. Dr. Pesenti received personal fees from Fresenius (Bad Homburg vor der Höhe, Germany), Getinge (Getinge, Sweden), and Baxter (Deerfield, Illinois), all outside the submitted work. The other authors declare no competing interests.

## Supplementary Material



## References

[1] Singer M Deutschman CS Seymour CW Shankar-Hari M Annane D Bauer M Bellomo R Bernard GR Chiche JD Coopersmith CM Hotchkiss RS Levy MM Marshall JC Martin GS Opal SM Rubenfeld GD van der Poll T Vincent JL Angus DC: The third international consensus definitions for sepsis and septic shock (Sepsis-3). JAMA. 2016; 315:801–10 26903338 10.1001/jama.2016.0287 PMC4968574

[2] Angus DC van der Poll T: Severe sepsis and septic shock. N Engl J Med. 2013; 369:840–51 23984731 10.1056/NEJMra1208623

[3] Saper CB Breder CD: The neurologic basis of fever. N Engl J Med. 1994; 330:1880–6 7832832 10.1056/NEJM199406303302609

[4] Astiz ME Tilly E Rackow ED Weil MH: Peripheral vascular tone in sepsis. Chest. 1991; 99:1072–5 2019159 10.1378/chest.99.5.1072

[5] Askanazi J Forse RA Weissman C Hyman AI Kinney JM: Ventilatory effects of the stress hormones in normal man. Crit Care Med. 1986; 14:602–5 3720307 10.1097/00003246-198607000-00002

[6] Weissman C Askanazi J Forse RA Hyman AI Milic-Emili J Kinney JM: The metabolic and ventilatory response to the infusion of stress hormones. Ann Surg. 1986; 203:408–12 3083792 10.1097/00000658-198604000-00012 PMC1251126

[7] Vaporidi K Akoumianaki E Telias I Goligher EC Brochard L Georgopoulos D: Respiratory drive in critically ill patients: Pathophysiology and clinical implications. Am J Respir Crit Care Med. 2020; 201:20–32 31437406 10.1164/rccm.201903-0596SO

[8] Linton RA Poole-Wilson PA Davies RJ Cameron IR: A comparison of the ventilatory response to carbon dioxide by steady-state and rebreathing methods during metabolic acidosis and alkalosis. Clin Sci Mol Med. 1973; 45:239–49 4522362 10.1042/cs0450239

[9] Tang GJ Kou YR Lin YS: Peripheral neural modulation of endotoxin-induced hyperventilation. Crit Care Med. 1998; 26:1558–63 9751593 10.1097/00003246-199809000-00024

[10] Magder S: Bench-to-bedside review: Ventilatory abnormalities in sepsis. Crit Care. 2009; 13:202 19216724 10.1186/cc7116 PMC2688092

[11] Montgomery AB Stager MA Carrico CJ Hudson LD: Causes of mortality in patients with the adult respiratory distress syndrome. Am Rev Respir Dis. 1985; 132:485–9 4037521 10.1164/arrd.1985.132.3.485

[12] Goligher EC Jonkman AH Dianti J Vaporidi K Beitler JR Patel BK Yoshida T Jaber S Dres M Mauri T Bellani G Demoule A Brochard L Heunks L: Clinical strategies for implementing lung and diaphragm-protective ventilation: Avoiding insufficient and excessive effort. Intensive Care Med. 2020; 46:2314–26 33140181 10.1007/s00134-020-06288-9 PMC7605467

[13] Viires N Sillye G Aubier M Rassidakis A Roussos C: Regional blood flow distribution in dog during induced hypotension and low cardiac output. Spontaneous breathing *versus* artificial ventilation. J Clin Invest. 1983; 72:935–47 6886012 10.1172/JCI111065 PMC1129259

[14] Delbove A Darreau C Hamel JF Asfar P Lerolle N: Impact of endotracheal intubation on septic shock outcome: A *post hoc* analysis of the SEPSISPAM trial. J Crit Care. 2015; 30:1174–8 26410680 10.1016/j.jcrc.2015.08.018

[15] Darreau C Martino F Saint-Martin M Jacquier S Hamel JF Nay MA Terzi N Ledoux G Roche-Campo F Camous L Pene F Balzer T Bagate F Lorber J Bouju P Marois C Robert R Gaudry S Commereuc M Debarre M Chudeau N Labroca P Merouani K Egreteau PY Peigne V Bornstain C Lebas E Benezit F Vally S Lasocki S Robert A Delbove A Lerolle N: Use, timing and factors associated with tracheal intubation in septic shock: A prospective multicentric observational study. Ann Intensive Care. 2020; 10:62 32449053 10.1186/s13613-020-00668-6 PMC7245631

[16] Frat JP Thille AW Mercat A Girault C Ragot S Perbet S Prat G Boulain T Morawiec E Cottereau A Devaquet J Nseir S Razazi K Mira JP Argaud L Chakarian JC Ricard JD Wittebole X Chevalier S Herbland A Fartoukh M Constantin JM Tonnelier JM Pierrot M Mathonnet A Béduneau G Delétage-Métreau C Richard JC Brochard L Robert R; FLORALI Study Group; REVA Network: High-flow oxygen through nasal cannula in acute hypoxemic respiratory failure. N Engl J Med. 2015; 372:2185–96 25981908 10.1056/NEJMoa1503326

[17] Mauri T Turrini C Eronia N Grasselli G Volta CA Bellani G Pesenti A: Physiologic effects of high-flow nasal cannula in acute hypoxemic respiratory failure. Am J Respir Crit Care Med. 2017; 195:1207–15 27997805 10.1164/rccm.201605-0916OC

[18] Mauri T Alban L Turrini C Cambiaghi B Carlesso E Taccone P Bottino N Lissoni A Spadaro S Volta CA Gattinoni L Pesenti A Grasselli G: Optimum support by high-flow nasal cannula in acute hypoxemic respiratory failure: Effects of increasing flow rates. Intensive Care Med. 2017; 43:1453–63 28762180 10.1007/s00134-017-4890-1

[19] Mauri T Grasselli G Suriano G Eronia N Spadaro S Turrini C Patroniti N Bellani G Pesenti A: Control of respiratory drive and effort in extracorporeal membrane oxygenation patients recovering from severe acute respiratory distress syndrome. Anesthesiology. 2016; 125:159–67 26999639 10.1097/ALN.0000000000001103

[20] Grieco DL Menga LS Raggi V Bongiovanni F Anzellotti GM Tanzarella ES Bocci MG Mercurio G Dell’Anna AM Eleuteri D Bello G Maviglia R Conti G Maggiore SM Antonelli M: Physiological comparison of high-flow nasal cannula and helmet noninvasive ventilation in acute hypoxemic respiratory failure. Am J Respir Crit Care Med. 2020; 201:303–12 31687831 10.1164/rccm.201904-0841OC

[21] Markovitz GH Colthurst J Storer TW Cooper CB: Effective inspired oxygen concentration measured via transtracheal and oral gas analysis. Respir Care. 2010; 55:453–9 20406513

[22] Roca O Caralt B Messika J Samper M Sztrymf B Hernández G García-de-Acilu M Frat JP Masclans JR Ricard JD: An index combining respiratory rate and oxygenation to predict outcome of nasal high-flow therapy. Am J Respir Crit Care Med. 2019; 199:1368–76 30576221 10.1164/rccm.201803-0589OC

[23] Derenne JP Couture J Iscoe S Whitelaw A Milic-Emili J: Occlusion pressures in men rebreathing CO_2_ under methoxyflurane anesthesia. J Appl Physiol. 1976; 40:805–14 931909 10.1152/jappl.1976.40.5.805

[24] Clark FJ von Euler C: On the regulation of depth and rate of breathing. J Physiol. 1972; 222:267–95 5033464 10.1113/jphysiol.1972.sp009797 PMC1331381

[25] Delorme M Bouchard PA Simon M Simard S Lellouche F: Physiologic effects of high-flow nasal cannula in healthy subjects. Respir Care. 2020; 65:1346–54 32291309 10.4187/respcare.07306

[26] Spinelli E Mauri T Beitler JR Pesenti A Brodie D: Respiratory drive in the acute respiratory distress syndrome: Pathophysiology, monitoring, and therapeutic interventions. Intensive Care Med. 2020; 46:606–18 32016537 10.1007/s00134-020-05942-6 PMC7224136

[27] Amoateng-Adjepong Y Jacob BK Ahmad M Manthous CA: The effect of sepsis on breathing pattern and weaning outcomes in patients recovering from respiratory failure. Chest. 1997; 112:472–7 9266886 10.1378/chest.112.2.472

[28] Noritomi DT Soriano FG Kellum JA Cappi SB Biselli PJ Libório AB Park M: Metabolic acidosis in patients with severe sepsis and septic shock: A longitudinal quantitative study. Crit Care Med. 2009; 37:2733–9 19885998 10.1097/ccm.0b013e3181a59165

[29] Huxtable AG Vinit S Windelborn JA Crader SM Guenther CH Watters JJ Mitchell GS: Systemic inflammation impairs respiratory chemoreflexes and plasticity. Respir Physiol Neurobiol. 2011; 178:482–9 21729770 10.1016/j.resp.2011.06.017 PMC3172820

[30] Doorduin J Leentjens J Kox M van Hees HW van der Hoeven JG Pickkers P Heunks LM: Effects of experimental human endotoxemia on diaphragm function. Shock. 2015; 44:316–22 26196838 10.1097/SHK.0000000000000435

[31] Mauri T Yoshida T Bellani G Goligher EC Carteaux G Rittayamai N Mojoli F Chiumello D Piquilloud L Grasso S Jubran A Laghi F Magder S Pesenti A Loring S Gattinoni L Talmor D Blanch L Amato M Chen L Brochard L Mancebo J; PLeUral pressure working Group (PLUG—Acute Respiratory Failure section of the European Society of Intensive Care Medicine): Esophageal and transpulmonary pressure in the clinical setting: Meaning, usefulness and perspectives. Intensive Care Med. 2016; 42:1360–73 27334266 10.1007/s00134-016-4400-x

[32] Plum F Posner JB: Blood and cerebrospinal fluid lactate during hyperventilation. Am J Physiol. 1967; 212:864–70 6024452 10.1152/ajplegacy.1967.212.4.864

[33] Gattinoni L Vasques F Camporota L Meessen J Romitti F Pasticci I Duscio E Vassalli F Forni LG Payen D Cressoni M Zanella A Latini R Quintel M Marini JJ: Understanding lactatemia in human sepsis: Potential impact for early management. Am J Respir Crit Care Med. 2019; 200:582–9 30985210 10.1164/rccm.201812-2342OC

[34] Vitacca M Bianchi L Zanotti E Vianello A Barbano L Porta R Clini E: Assessment of physiologic variables and subjective comfort under different levels of pressure support ventilation. Chest. 2004; 126:851–9 15364766 10.1378/chest.126.3.851

[35] Baydur A Behrakis PK Zin WA Jaeger M Milic-Emili J: A simple method for assessing the validity of the esophageal balloon technique. Am Rev Respir Dis. 1982; 126:788–91 7149443 10.1164/arrd.1982.126.5.788

[36] Shi ZH de Vries H de Grooth HJ Jonkman AH Zhang Y Haaksma M van de Ven PM de Man AAME Girbes A Tuinman PR Zhou JX Ottenheijm C Heunks L: Changes in Respiratory muscle thickness during mechanical ventilation: Focus on expiratory muscles. Anesthesiology. 2021; 134:748–59 33711154 10.1097/ALN.0000000000003736

[37] Hussain SN Roussos C: Distribution of respiratory muscle and organ blood flow during endotoxic shock in dogs. J Appl Physiol (1985). 1985; 59:1802–8 4077788 10.1152/jappl.1985.59.6.1802

[38] Hussain SN Graham R Rutledge F Roussos C: Respiratory muscle energetics during endotoxic shock in dogs. J Appl Physiol (1985). 1986; 60:486–93 3949653 10.1152/jappl.1986.60.2.486

[39] CAMPBELL EJ DINNICK OP HOWELL JB: The immediate effects of elastic loads on the breathing of man. J Physiol. 1961; 156:260–73 13690227 10.1113/jphysiol.1961.sp006674 PMC1359884

