# Supplementary material for: Respiratory Drive in Patients with Sepsis and Septic Shock: Modulation by High-flow Nasal Cannula
Source: Anesthesiology. 2021 Oct 13;135(6):1066–75. doi: 10.1097/ALN.0000000000004010 (PMC10348628; doi:10.1097/ALN.0000000000004010)
Supplement: Supplementary file 1 [file aln-135-1066-s001.docx]

**Respiratory drive in patients with sepsis and septic shock: modulation by high flow nasal cannula**

T. Mauri et al.

**Online Supplement**

# Methods

## Physiological data

EIT devices, acquisition systems, and software for offline analyses used in the 3 study centres are provided in Table A1. All physiological data were then centralized to create a single dataset for statistical analysis.

## Respiratory support

Respiratory support provided during the LFO phases is reported in Table A2.

## Esophageal pressure tracings

All esophageal pressure tracings were analyzed by two independent researchers. All data were computed as the mean of 10 representative respiratory cycles recorded towards the end of each study step.

Poor quality tracings, due to esophageal probe misplacement or balloon deflation were discarded after analysis and discussion by 3 authors (TM, BP and SS). Tracings were deemed of poor quality in 4 (16%) patients (examples in Figure A1a and Figure A1b).

Variables measured from esophageal pressure waveforms are summarized in the Figure A2.

## Tidal volume calibration

In this study, EIT data is expressed in milliliters. To convert from impedance, the authors applied the same calibration factor to all patients, derived from a previously published cohort of hypoxemic patients (Mauri et al., Intensive Care Med 2017). The calibration factor from arbitrary units to milliliters was 0.24 mL/AU.

# Results

## Subgroups analysis

Two additional analyses were performed in the sub-groups of patients with high quality valid Pes tracings (n = 21, Table A3) and with PaO_2_/FiO_2_ ratio >200 (n = 21, Table A4). Data and differences between study phases of respiratory drive and effort, lung volumes, gas exchange and hemodynamics were similar to the global population (see Table 2 in the main text).

## Interaction between respiratory effort and drive and comfort

There was no correlation between the comfort reported by the patient (VAS) and the markers of effort (ΔPes) and drive (P_0.5_). These results are provided in the Figure A3.

## Correlation between respiratory drive and effort

There was a correlation between each measure of respiratory drive and effort (ΔPes). These results are reported in the Figure A4.

## Predictors of larger improvement of effort by HFNC

The authors described significant correlation between respiratory rate and ΔPes measured during the LFO-baseline step and the decrease of ΔPes during the HFNC step (Figure A5).

| **Centre** | **Pes acquisition** | **Pes analysis** | **EIT acquisition** | **EIT analysis** |
| --- | --- | --- | --- | --- |
|  |  |  |  |  |
| **Maggiore Policlinico Hospital**  Milan, Italy | Colligo (Elekton, Milano, Italy) | Acqknowledge 4.0 (Biopac Systems, Goleta, CA, USA) | Pulmovista (Draeger, Lubeck, Germany) | EITdiag v6.3 ((Draeger, Lubeck, Germany)  MATLAB (Mathworks, Natick, MA, USA) |
|  |  |  |  |  |
| **Sant'Anna University Hospital**  Ferrara, Italy | ICU lab v3.1 (KleisTEK Engineering, Bari, Italy) | Acqknowledge 4.0 (Biopac Systems, Goleta, CA, USA) | Swisstom BB2 (SenTec, Landquart, Switzerland) | Ibex (SenTec, Landquart, Switzerland) |
|  |  |  |  |  |
| **IRCCS Policlinico Universitario A. Gemelli**  Rome, Italy | ICU lab v3.1 (KleisTEK Engineering, Bari, Italy) | Acqknowledge 4.0 (Biopac Systems, Goleta, CA, USA) | n.a. | n.a. |
|  |  |  |  |  |

**Table A1. Devices and software used for esophageal pressure (Pes) and electrical impedance tomography (EIT) analysis, in each participating center.**

| **Pt.** | **Device** | **O_2_ Flow**  **L.min^-1^** |
| --- | --- | --- |
| **1** | LFNC | 10 |
| **2** | LFNC | 2 |
| **3** | LFNC | 2 |
| **4** | LFNC | 0.5 |
| **5** | LFNC | 4 |
| **6** | LFNC | 1 |
| **7** | LFNC | 4 |
| **8** | LFNC | 4 |
| **9** | LFNC | 4 |
| **10** | LFNC | 4 |
| **11** | Ambient air | 0 |
| **12** | LFNC | 2 |
| **13** | Ambient air | 0 |
| **14** | Venturi mask | 8 |
| **15** | LFNC | 4 |
| **16** | Venturi mask | 8 |
| **17** | LFNC | 4 |
| **18** | LFNC | 4 |
| **19** | LFNC | 4 |
| **20** | LFNC | 4 |
| **21** | Venturi mask | 8 |
| **22** | LFNC | 3 |
| **23** | Venturi mask | 15 |
| **24** | Venturi mask | 15 |
| **25** | Ambient air | 0 |

**Table A2. Individual data on the device used during the low flow oxygenation (LFO) study phases.** *LFNC = low-flow nasal cannula, Pt. = patient.*

|  | **LFO-baseline** | **HFNC** | **LFO-end** | **ANOVA *p* value** |
| --- | --- | --- | --- | --- |
|  |  |  |  |  |
| **Respiratory effort** |  |  |  |  |
| ΔPes, cmH_2_O | 8.0 [6.0-11.5] | 5.5 [4.5-8.0] † | 7.5 [6.0-12.6] | **< .001** |
| PTP_Pes_, cmH_2_O.s.min^-1^ | 224 [184-300] | 140 [84-192] † | 210 [174-275] | **< .001** |
|  |  |  |  |  |
| **Respiratory drive** |  |  |  |  |
| P_0.5_, cmH_2_O | 6.0 [4.4-9.0] | 4.3 [3.5-6.6] † | 6.6 [4.9-10.7] | **< .001** |
| ΔPes/Δt, cmH_2_O.s^-1^ | 9.0 [5.4-13.0] | 5.7 [4.6-8.8] † | 10.0 [6.2-14.2] | **< .001** |
| VT/Ti, mL.s^-1^ | 551 [466-682] | 489 [424-593] * | 533 [421-652] | **.009** |
|  |  |  |  |  |
| **Lung volumes by EIT** |  |  |  |  |
| VT, mL | 504 [327-650] | 525 [383-725] | 526 [373-726] | .360 |
| Respiratory rate, min^-1^ | 23 [19-29] | 20 [14-26] † | 22 [18-26] | **.008** |
| MV, L.min^-1^ | 11.6 [9.0-15.0] | 10.5 [8.2-14.5] | 11.5 [7.7-14.7] | .191 |
| VT/ΔPes, mL.cmH_2_O^-1^ | 63 [36-105] | 83 [71-144] † | 65 [41-114] | **.003** |
| VT_NDEP_/_DEP_ | 1.21 [0.71-1.66] | 1.11 [0.74-1.66] | 1.28 [0.83-1.64] | .081 |
|  |  |  |  |  |
| **Gas exchange** |  |  |  |  |
| pH | 7.43 [7.39-7.46] | 7.43 [7.38-7.47] | 7.42 [7.40-7.47] | .563 |
| PaCO_2_, mmHg | 33.5 [30.6-41.7] | 33.9 [29.2-39.2] | 34.0 [30.4-40.7] | .267 |
| PaO_2_/FiO_2_, mmHg | 257 [211-330] | 315 [247-363] | 304 [244-367] | .142 |
| SaO_2_, % | 96 [94-97] | 96 [94-96] | 96 [95-97] | .371 |
|  |  |  |  |  |
| **Hemodynamics** |  |  |  |  |
| MAP, mmHg | 83 [71-97] | 80 [70-92] | 76 [67-95] | .153 |
| HR, bpm | 93 [78-117] | 100 [84-111] | 91 [77-115] | .232 |
|  |  |  |  |  |

**Table A3. Physiological effects of high flow nasal cannula (HFNC) in the sub-group of patients with valid high quality esophageal pressure tracings (n=21).** Dunnett’s post-hoc tests: * p<0.05 vs. LFO-Baseline, † p<0.005 vs. LFO-Baseline.

*ΔPes: negative esophageal pressure swing, EIT: electrical impedance tomography, FiO_2_: inspired dioxygen fraction, HR: heart rate, LFO: low flow oxygen, MAP: mean arterial pressure, MV: minute ventilation, PaCO_2_: arterial carbon dioxide partial pressure, PaO_2_: arterial dioxygen partial pressure, PTP_Pes_: pressure-time product, RR: respiratory rate, Ti: inspiratory time, VT: tidal volume, VT_NDEP/DEP_: non-dependent on dependent regions tidal volume ratio, SaO_2_: arterial dioxygen saturation.*

|  | **LFO-baseline** | **HFNC** | **LFO-end** | **ANOVA *p* value** |
| --- | --- | --- | --- | --- |
|  |  |  |  |  |
| **Respiratory effort** |  |  |  |  |
| ΔPes, cmH_2_O | 7.5 [5.7-12.3] | 5.6 [4.9-9.0] * | 7.5 [5.8-12.3] | **.004** |
| PTP_Pes_, cmH_2_O.s.min^-1^ | 224 [141-302] | 140 [85-213] † | 210 [123-277] | **.007** |
|  |  |  |  |  |
| **Respiratory drive** |  |  |  |  |
| P_0.5_, cmH_2_O | 4.9 [4.1-9.0] | 4.4 [3.8-6.6] | 6.6 [4.9-10.0] | **.003** |
| ΔPes/Δt, cmH_2_O.s^-1^ | 9.0 [5.4-13.0] | 5.8 [4.9-8.8] | 10.0 [6.4-13.4] | **.010** |
| VT/Ti, mL.s^-1^ | 527 [468-658] | 490 [369-592] * | 489 [378-642] | **.035** |
|  |  |  |  |  |
| **Lung volumes by EIT** |  |  |  |  |
| VT, mL | 534 [339-718] | 600 [443-737] | 612 [387-757] | .829 |
| Respiratory rate, min^-1^ | 22 [18-26] | 19 [14-24] † | 22 [15-25] | **.002** |
| MV, L.min^-1^ | 12.0 [9.7-15.8] | 11.1 [8.7-15.0] | 12.3 [8.8-15.5] | .117 |
| VT/ΔPes, mL.cmH_2_O^-1^ | 82 [36-127] | 84 [67-170] * | 73 [42-130] | **.022** |
| VT_NDEP_/_DEP_ | 1.24 [0.86-1.57] | 1.21 [0.80-1.52] | 1.37 [0.85-1.55] | .117 |
|  |  |  |  |  |
| **Gas exchange** |  |  |  |  |
| pH | 7.43 [7.40-7.46] | 7.44 [7.39-7.47] | 7.41 [7.41-7.46] | .647 |
| PaCO_2_, mmHg | 33.5 [29.5-38.5] | 33.4 [29.2-37.8] | 34.0 [30.4-37.5] | .176 |
| PaO_2_/FiO_2_, mmHg | 296 [239-371] | 348 [291-375] | 318 [267-371] | .187 |
| SaO_2_, % | 96 [95-97] | 96 [94-96] | 96 [95-96] | .438 |
|  |  |  |  |  |
| **Hemodynamics** |  |  |  |  |
| MAP, mmHg | 81 [71-98] | 80 [71-89] | 76 [72-91] | .351 |
| HR, bpm | 105 [86-121] | 103 [86-118] | 102 [83-118] | .526 |
|  |  |  |  |  |

**Table A4. Physiological effects of high flow nasal cannula (HFNC) in the sub-group of patients with PaO_2_/FiO_2_ ratio >200 mmHg (n=21).** Dunnett’s post-hoc tests: * p<0.05 vs. LFO-Baseline, † p<0.005 vs. LFO-Baseline.

*ΔPes: negative esophageal pressure swing, EIT: electrical impedance tomography, FiO_2_: inspired dioxygen fraction, HR: heart rate, LFO: low flow oxygen, MAP: mean arterial pressure, MV: minute ventilation, PaCO_2_: arterial carbon dioxide partial pressure, PaO_2_: arterial dioxygen partial pressure, PTP_Pes_: pressure-time product, RR: respiratory rate, Ti: inspiratory time, VT: tidal volume, VT_NDEP/DEP_: non-dependent on dependent regions tidal volume ratio, SaO_2_: arterial dioxygen saturation.*

**
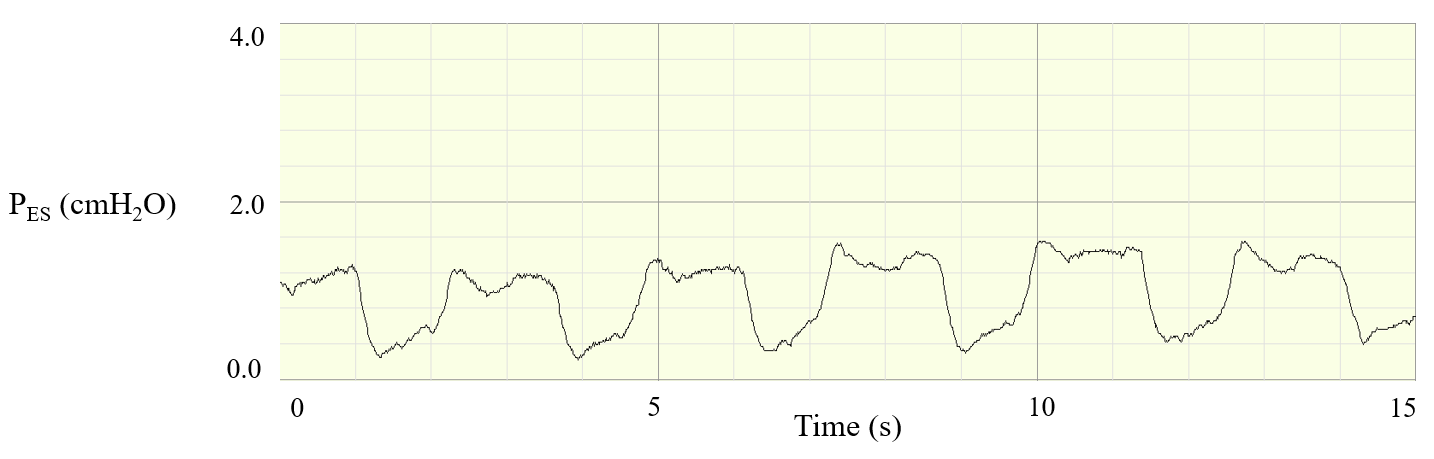
**

**Figure A1a. Example of a rejected esophageal pressure tracing, probably due to balloon deflation.** Note the low baseline level and minimal amplitude of the negative swings.

**
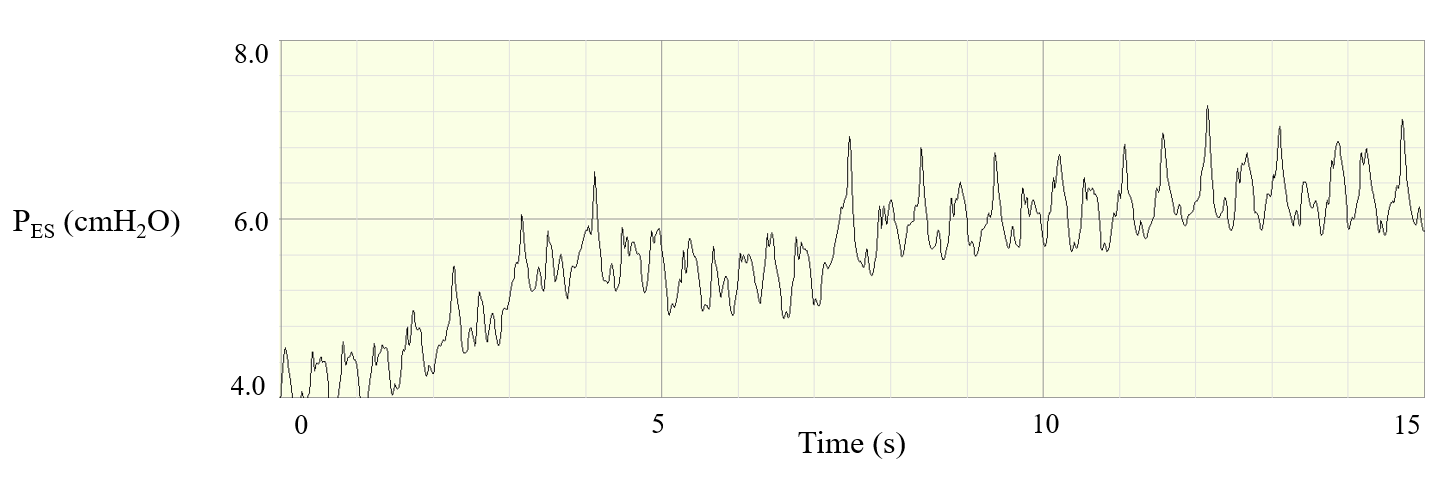
**

**Figure A1b. Example of a rejected esophageal pressure tracing, probably due to balloon misplacement.** Note the fluctuations of baseline and lack of negative swings.

**
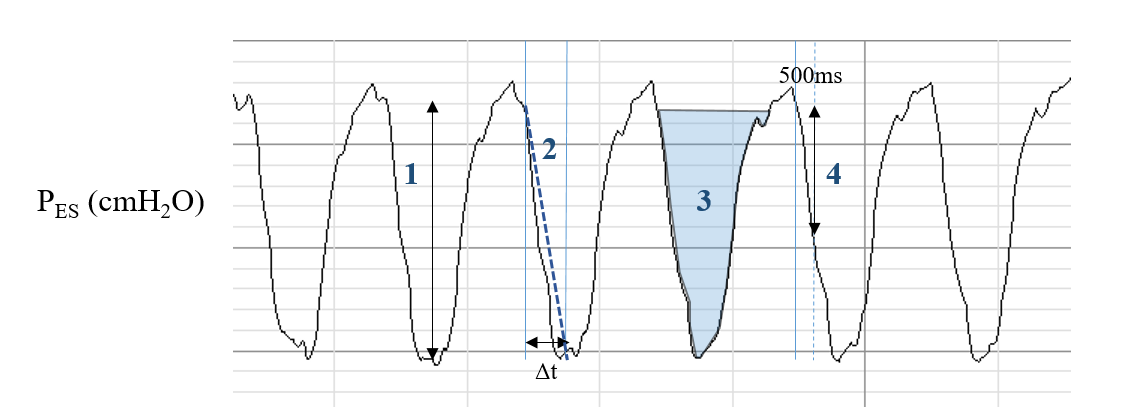
**

**Figure A2. Example of esophageal pressure tracings, with measured variables.**

(1) and (3) Respiratory effort: ΔPes and PTP_Pes_

(2) and (4) Respiratory drive: ΔPes/Δt and P_0.5_

**
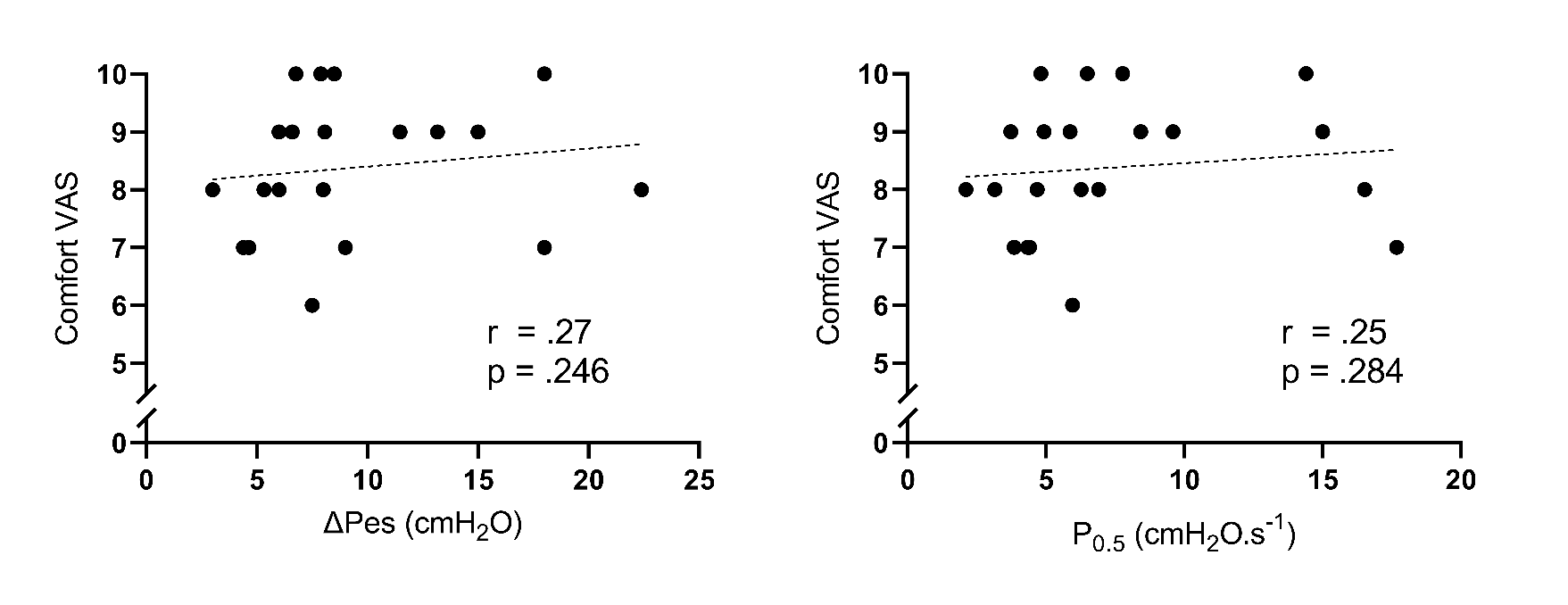
**

**Figure A3. Correlations between respiratory effort (ΔPes), drive (P_0.5_), and the VAS comfort scale.** VAS: Visual analogic scale.

**
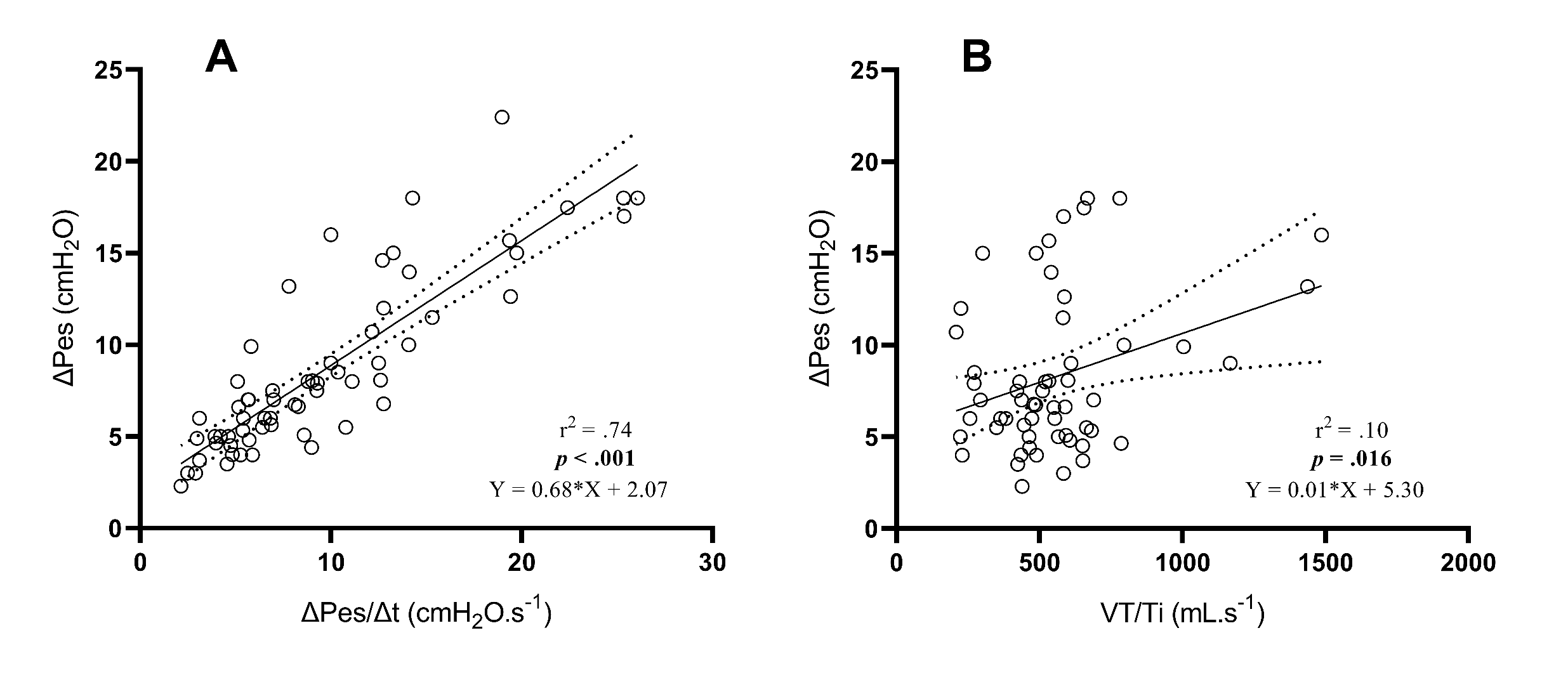
**

**Figure A4. Correlations between measures of respiratory drive and ΔPes.** Data from the three study phases were gathered to provide the linear regression analysis (n = 63). *ΔPes: negative esophageal pressure swing, Δt. time from inspiratory start to minimum Pes, Ti: inspiratory time, VT: tidal volume.*

**
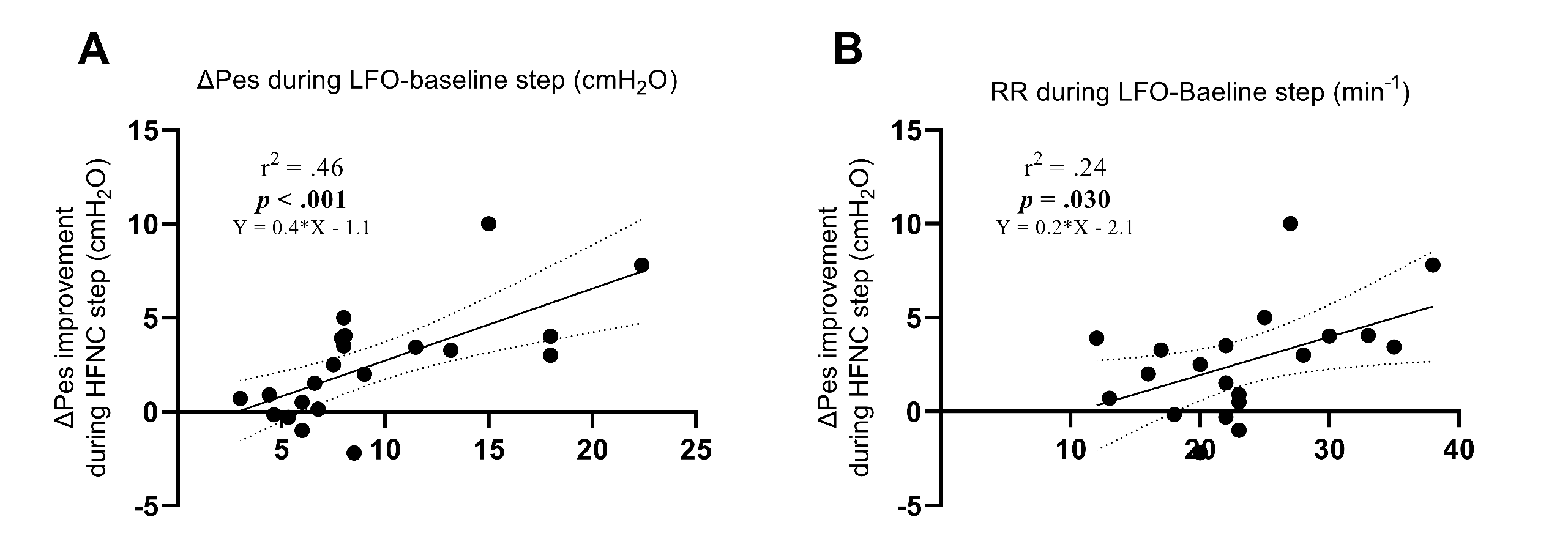
**

**Figure A5. Correlation between ΔPes (A) and respiratory rate (RR, B) measured during LFO-baseline and reduction of ΔPes during the HFNC step.** *ΔPes: negative esophageal pressure swing.*
